# Impact of COVID-19 on the treatment of depressive patients in Germany–a gap in care for the mentally ill?

**DOI:** 10.3389/fpsyt.2023.1198632

**Published:** 2023-09-27

**Authors:** Mareike Aichholzer, Carmen Schiweck, Carmen Uckermark, Tirage Hamzehloiya, Christine Reif-Leonhard, Rejane Golbach, Andreas Reif, Sharmili Edwin Thanarajah

**Affiliations:** ^1^Department for Psychiatry, Psychosomatic Medicine and Psychotherapy, University Hospital Frankfurt, Goethe University, Frankfurt, Germany; ^2^Department of Medicine, Institute of Biostatistics and Mathematical Modelling, Goethe University, Frankfurt, Germany

**Keywords:** depression, COVID-19, pandemic, Germany, psychiatry

## Abstract

**Background:**

The COVID-19 pandemic led to a higher incidence of depression and a worsening of psychiatric conditions, while pre-existing constraints of the healthcare system and safety regulations limited psychiatric care.

**Aims:**

We investigated the impact of the pandemic on the clinical care of patients with a single episode (SE-MDD) or major depressive disorder (MDD) in Germany.

**Methods:**

Nationwide inpatient data were extracted from the German Institute for Hospital Remuneration System for 2020 and 2021 (depression data) and the Robert Koch Institute (COVID-19 incidence). Changes in inpatients were tested with linear regression models. Local cases of depression in our department compared to 2019 were explored with one-way ANOVA and Dunnett's test.

**Results:**

Across Germany, the inpatient numbers with both SE-MDD and MDD declined by more than 50% during three out of four COVID-19 waves. Higher COVID-19 incidence correlated with decreased inpatient numbers. In our department, fewer MDD inpatients were treated in 2020 (adj. *p* < 0.001) and 2021 (adj. *p* < 0.001) compared to 2019, while the number of SE-MDD inpatients remained stable. During this period fewer elective and more emergency inpatients were admitted. In parallel, MDD outpatient admissions increased in 2021 compared to 2019 (adj. *p* = 0.002) and 2020 (adj. *p* = 0.003).

**Conclusion:**

During high COVID-19 infection rates, MDD patients received less inpatient care, which might cause poor outcomes in the near future. These data highlight the necessity for improved infrastructure in the in- and outpatient domains to facilitate accessibility to adequate care.

## 1. Introduction

On the 30^th^ of January 2020, the World Health Organization classified the “severe acute respiratory syndrome coronavirus 2 (SARS-CoV-2)” as a “public health emergency of international concern” (1). In Germany, the first COVID-19 infection was confirmed on the 27^th^ of January 2020 (2). The resulting COVID-19 pandemic and the restrictions on daily life have been a major impact on mental health: The restrictions that were required to prevent the virus from spreading including quarantine, self-isolation, social distancing, and school closures all over the world represented a major challenge for mental health (3). The impact of these interventions on the course of somatic diseases such as cancer has been examined in numerous studies (4–7). For example, the number of screening investigations for breast cancer has significantly decreased in the United Kingdom leading to a later diagnosis with a worse outcome and a higher economic burden for society (5). It is highly conceivable that similar outcomes are to be expected for psychiatric care. However, studies on implications for psychiatric care with a large number of patients are still missing.

Studies from various countries have shown an increased mental health burden during the pandemic (8). In this context, depression is particularly relevant. First of all, it is one of the leading causes of years lived with disabilities worldwide leading to a critical health-economic burden (9, 10). In Germany, roughly 5.3 million people suffer from depression, with women being twice to thrice times more affected than men (11). Second, there is increasing evidence, that the number of newly diagnosed cases of depression and anxiety has increased worldwide (12, 13). Increased depressive symptoms has not only been reported in the elderly (14) but also in the adolescent population (15).

In general, a worsening of symptoms in psychiatric patients was observed during the pandemic regardless if the patients were infected with the virus itself or not (16). Whether the COVID-19 pandemic has led to increased rates of suicidal behavior is still under debate, the findings differ depending on the countries, sample, and time period, that has been investigated (17, 18).

The abovementioned increase in mental health burden raises the question if the healthcare system has adequately addressed the increased demand for specialized psychiatric and psychological therapy during the pandemic. In Germany, psychiatric hospitals are obliged to tend for psychiatric patients who live within an officially defined area around the hospital. Beside the inpatient treatment, hospitals offer daycare (being treated in the hospital during the day but spending the night at home) and outpatient treatment. Moreover, psychiatric patients are treated by general practitioners and psychiatrists in private practices. However, a lack of funding, reduction of inpatient treatment capacities (19), scarcity of outpatient treatment, especially in rural areas (20), and the fact that only one-fifth of all psychiatric patients are in treatment (21) point toward an overburdened German mental health care system well before the pandemic.

First data from Germany showed a decline in the number of psychiatric emergency hospital admissions during the first wave of the COVID-19 outbreak when compared to the period before the outbreak (22). With this study, we aimed to investigate whether in- and outpatient care of patients with a first depressive episode (SE-MDD) and recurrent depressive disorder (MDD) were impacted during the COVID-19 pandemic in Germany.

## 2. Methods

### 2.1. Data acquisition

In Germany, all hospitals are legally obliged to report the ICD-10 diagnosis of inpatients in an anonymized form to the German Institute for Hospital Remuneration System (“Institut für das Entgeltsystem im Krankenhaus,” InEK). For this retrospective analysis, nationwide data was extracted from the InEK, which was open to the public for the years 2020 and 2021. The case numbers were retrieved in duplicate by CU and TH and afterward checked for differences by MA. Since the German-wide data neither allow comparison with pre-COVID years nor give any information about outpatient care during the pandemic, we additionally investigated anonymized cases of in- and outpatients at our Department of Psychiatry, Psychosomatic Medicine and Psychotherapy at the Goethe University Frankfurt (GUF), for the years 2020 and 2021 in comparison to the pre-pandemic year 2019. The COVID-19 infection rates of Germany and Frankfurt for the respective time periods were obtained from the publicly available platform of the Robert-Koch-Institute. Since exclusively anonymized data were used, no vote of an ethics committee was required. The study was conducted in accordance with the Helsinki Declaration (revised in 1989).

### 2.2. Inclusion criteria

All inpatients admitted to any German hospital between January 1, 2020, and December 31, 2021, for the following main diagnoses were included in the analysis irrespective of disease severity and comorbidities: major depressive disorder–single episode (F32, SE-MDD), major depressive disorder–recurrent (F33, MDD). Patients who received treatment in daycare–spending the night at home but being on the wards during the day–were considered to be “inpatients.”

In the second analysis, we included all patients, who were admitted to in- and outpatient care at GUF between January 2019 and December 2021 with the following main diagnoses: major depressive disorder–recurrent (F33) and major depressive disorder–single episode (F32). Only limited demographic information (sex, age) was available for the patients treated at GUF since the data was anonymized.

### 2.3. Statistical analysis

Statistical data analysis was performed in Rstudio (vers. 2022.7.0.548) (23) and GraphPad Prism (24).

#### 2.3.1. Nationwide inpatient numbers

Linear regression was conducted with the “*nlme*” package (25) for the influence of COVID-19-incidence on nationwide inpatient numbers (diagnoses: F32, F33) in the years 2020 and 2021; analyses accounted for the autocorrelation of numbers between subsequent months in the time series. In addition, we examined the monthly admission rates of the whole cohort and separately for both sexes for the entire period (January 2020–December 2021) in relation to the national COVID-19-rates and quantified the decline during the peak of each wave in comparison to the period 3 months earlier to investigate the specific impact of the COVID-19-waves on the admission numbers.

#### 2.3.2. In- and outpatient numbers at GUF

Following visual inspection and a Shapiro-Wilk test to ensure a normal distribution, the monthly in- and quarterly outpatient numbers at GUF for the years 2019 (pre-COVID year), 2020, and 2021 were compared using a one-way ANOVA. Dunnett's multiple comparisons test was conducted *post-hoc* between all 3 years and multiplicity adjusted *P* values are reported. SE-MDD inpatient numbers were not normally distributed and therefore compared with the Kruskal-Wallis test. Next, the percent change of the quarterly admission rates for 2020 and 2021 compared to the equivalent time period in 2019 (Δadmissions) was examined in relation to the COVID-19-rates in Frankfurt. Furthermore, the percentage between emergency admissions and planned elective inpatient admissions to GUF of the years 2019–2021 was reported.

Statistical significance was indicated at a level of *p* < 0.05. All figures were created using GraphPad Prism (24).

## 3. Results

### 3.1. Inpatient admissions across Germany with the primary diagnosis of SE-MDD and MDD declined during each COVID-19 wave

**Figure 1:**
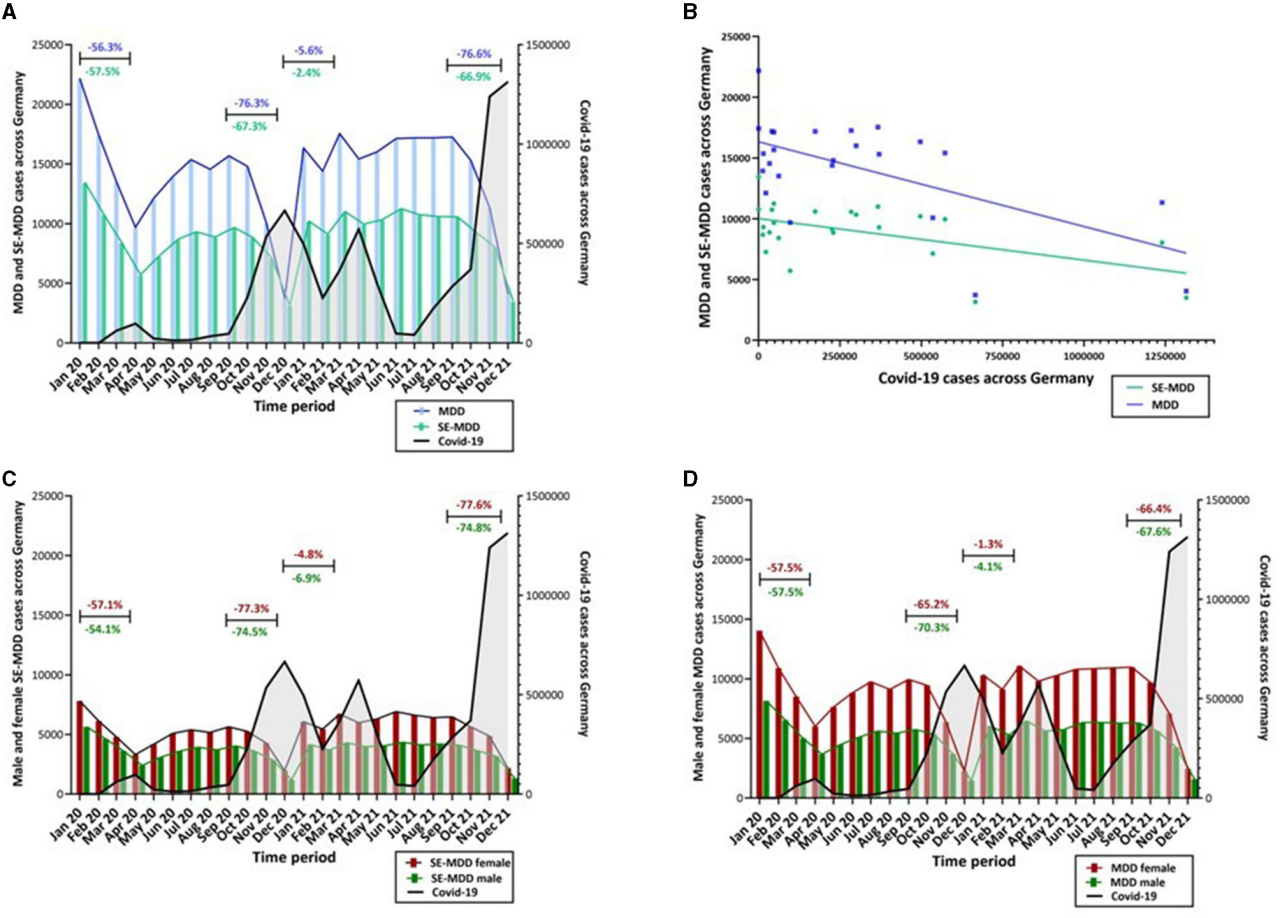
**(A)** During the period of January 2020 and December 2021 the nationwide number of inpatients with the primary diagnosis of MDD (F33, blue) and SE-MDD (F32, green) showed a clear drop during each COVID-19 wave (April 2020, December 2020, April 2021 and December 2021). The black line indicates the COVID-19 incidence across Germany. **(B)** Linear regression analysis showed that a rise in COVID-19 cases was associated with a significant decline in German wide inpatient admissions with SE-MDD (*F*_(1, 22)_ = 6.97, *p* = 0.015, ß = −0.004) and MDD (*F*_(1, 22)_ = 10,42, *p* = 0.004, ß = −0.008). **(C)** Over the years 2020 and 2021 a clear drop of female (red) and male (green) SE-MDD patients was noticeable during each COVID-19 wave. The sex difference (more female than male patients) remained stable during the 2 years. **(D)** Both sexes, female (red) and male (green), with MDD showed a drop during each Covid-19 wave, while more women were treated. The ratio of female and male patients was unchanged during the period of January 2020 and December 2021.

According to the InEK platform, 558,326 patients with the primary diagnosis of SE-MDD or MDD were admitted to psychiatric hospitals across Germany between the 1^st^ of January 2020 and the 31^st^ of December 2021. The demographic data of these patients are displayed in Table 1. In the abovementioned period, the COVID-19 rates reached four peaks (Figure 1A) in Germany. The peaks of new, registered COVID-19 infections were (1) April 2020 (97,206 cases), (2) December 2020 (665,868 cases), (3) April 2021 (572,724 cases) and (4) December 2021 (1,313,609 cases). This is in line with the four COVID-19 waves, that were officially registered in Germany (26, 27).

**Table 1:** Demographic data of inpatients treated across Germany in 2020 and 2021 with a SE-MDD or MDD diagnosis.

		**SE-MDD**		**MDD**	
**Year**		**2020**	**2021**	**2020**	**2021**
Cases (*n*)		101,356	114,655	196,293	179,202
Sex (*n*)	Male	42,357	45,048	73,310	65,824
	Female	58,979	69,561	122,944	113,335
	Diverse	10	23	20	36
	Unknown	10	23	20	6
Average length of stay (*days*)		29	31	32	2
Age groups (*years*)	3–5	10	0	0	0
	6–9	152	126	27	0
	10–15	10,156	14,561	1,839	661
	16–17	7,206	9,310	1,872	1,210
	18–29	21,802	25,362	33,888	34,115
	30–39	13,055	14,412	28,170	24348
	40–49	12,335	12,589	32,253	28,198
	50–54	8,554	8,542	24,901	21,312
	55–59	8,706	9,276	25,999	23,343
	60–64	5,595	6,272	16,193	15,444
	65–74	5,980	6,317	15,689	15,134
	75–79	3,112	2,843	7,306	5,737
	>80	4,703	5,033	8,185	7,573

During each wave, we identified a clear decline in inpatient numbers in comparison to the period 3 months earlier (Figure 1A): During the first wave, in April 2020, 56.3% fewer inpatients with MDD and 57.5% fewer patients with SE-MDD were hospitalized. During the peak of the second COVID-19 wave, the number of admissions dropped by 67.3 % for SE-MDD and 76.2% for MDD. In the third wave in April 2021, only 2.4% fewer patients with a diagnosis of SE-MDD and 5.6% fewer patients with MDD were admitted to a psychiatric ward. During the fourth wave, in December 2021, however, the number of admissions declined by 66.9% for SE-MDD and by 76.6% for MDD.

In general, more female patients were admitted with both SE-MDD and MDD. During the abovementioned waves, the decline in male and female patients followed the same patterns for both conditions (Figures 1C, D).

Across the whole time period from 01/2020 till 12/2021 the number of COVID-19 cases predicted a significant decline in the number of admissions with SE-MDD (*F*_(1, 22)_ = 6.97, *p* = 0.015, ß = −0.004) and MDD (*F*_(1, 22)_ = 10.42, *p* = 0.004, ß = −0.008) (Figure 1B).

### 3.2. The inpatient admissions with MDD declined during the pandemic, while admissions with SE-MDD remained stable at GUF

**Figure 2:**
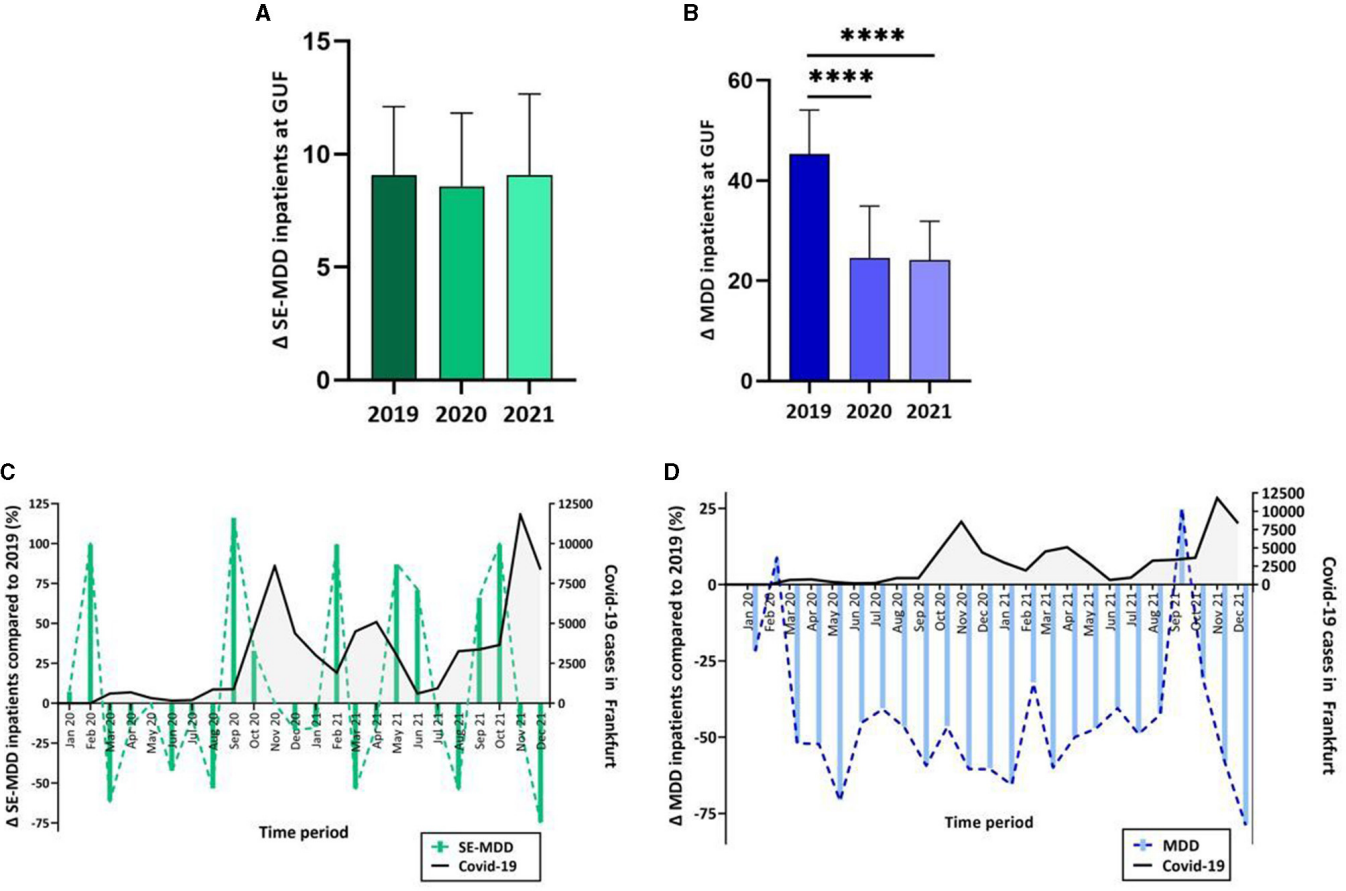
**(A)** The monthly inpatient number with SE-MDD at GUF showed no significant change over the years 2019, 2020, and 2021, whereas **(B)** the numbers of MDD inpatients at GUF showed a significant decline between the years 2019 and 2021 as well as 2019 and 2020. **(C)** Admissions with SE-MDD (green) declined during each COVID-19 wave in Frankfurt. **(D)** Fewer patients were admitted with MDD (blue) during the years 2020 and 2021 compared to the year 2019. Admission rates are depicted as a percentage change in 2020 and 2021 relative to each month in the pre-COVID year 2019. ****multiplicity adj. *p* < 0.0001.

Next, we explored the number of inpatients with SE-MDD and MDD admitted to our psychiatry department at GUF during 2020 and 2021 in comparison to the pre-COVID year (Table 2). While we found no significant change in the number of SE-MDD between these years (Kruskal-Wallis test, *H*_(3)_ = 0.25, *p* = 0.882) (Figure 2A), we found a significant difference in inpatient numbers with MDD between 2019, 2020, and 2021 (one-way ANOVA, *F*_(2, 33)_ = 21.32, *p* < 0.001) (Figure 2B). In the *post-hoc* comparison, the inpatient numbers were significantly lower in 2020 (multiplicity adj. *p* < 0.001, 95% C.I. = 12.20, 29.30) as well as 2021 (multiplicity adj. *p* < 0.001, 95% C.I. = 12.54, 29.63) compared to 2019. Independently of the start of the pandemic more female than male patients were admitted over the years 2019, 2020 and 2021 (Table 2).

**Table 2:** Demographic data of inpatients at GUF in 2019, 2020, and 2021 with SE-MDD and MDD.

**Year**	**SE-MDD (*n*)**	**MDD (*n*)**	**Male (%)**	**Female (%)**
2019	109	542	47	53
2020	103	295	45	55
2021	109	289	47	53

The percent change of monthly inpatient numbers in 2020 and 2021 compared to the corresponding period in 2019 (Δadmissions) showed opposing trends for SE-MDD and MDD: For SE-MDD, we observed a decline in Δadmissions during each wave and an increase in Δadmissions between the waves (Figure 2C). In contrast, fewer patients were hospitalized with MDD during the whole period between January 2020 and December 2021 in comparison to the corresponding time in 2019 (except for February 2020 and September 2021) (Figure 2D).

### 3.3. Shift toward more emergency admissions to psychiatric wards at GUF since the start of the pandemic

**Figure 3:**
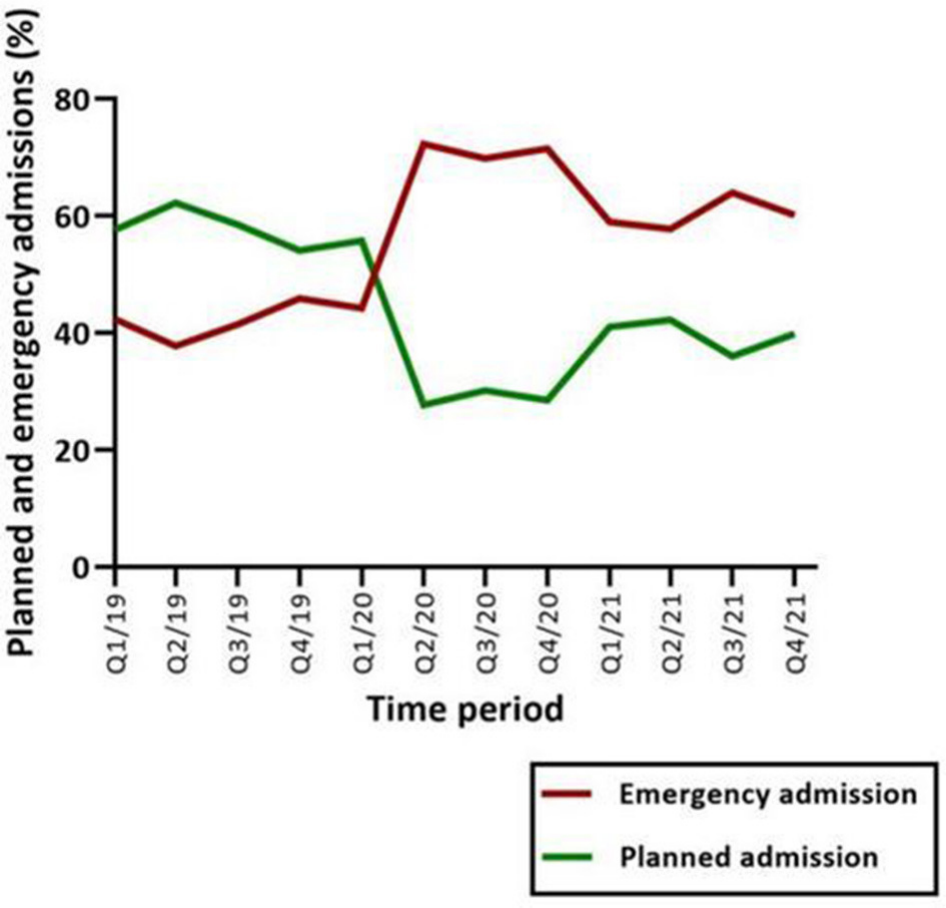
With the onset of the pandemic, the ratio of planned to emergency admissions of psychiatric patients reversed. Since the pandemic, the rate of emergency admissions has remained significantly higher. Q1-4 = quarter 1-4.

The ratio between planned, elective, and emergency admissions changed during the pandemic. A shift toward a higher rate of emergency admissions was evident across all psychiatric diagnoses. In the second quarter of 2020 the numbers of overall emergency admissions (72.29%) exceeded by far the elective admissions (27.71%), whereas in the fourth quarter of 2019, 54.13% of the admissions were planned and 45.87% were admitted on an emergency basis (Figure 3). Regarding patients with depression (MDD and SE-MDD), planned admission dropped by 45.37% in 2020 and 50.93% in 2021, while the number of emergency admission increased by 33.80% in 2021 and 43.66% in comparison to 2019.

### 3.4. Increase in outpatient numbers of SE-MDD is bound to each COVID-19-wave, while MDD outpatients show a significant increase over the years 2019, 2020, and 2021 at GUF

**Figure 4:**
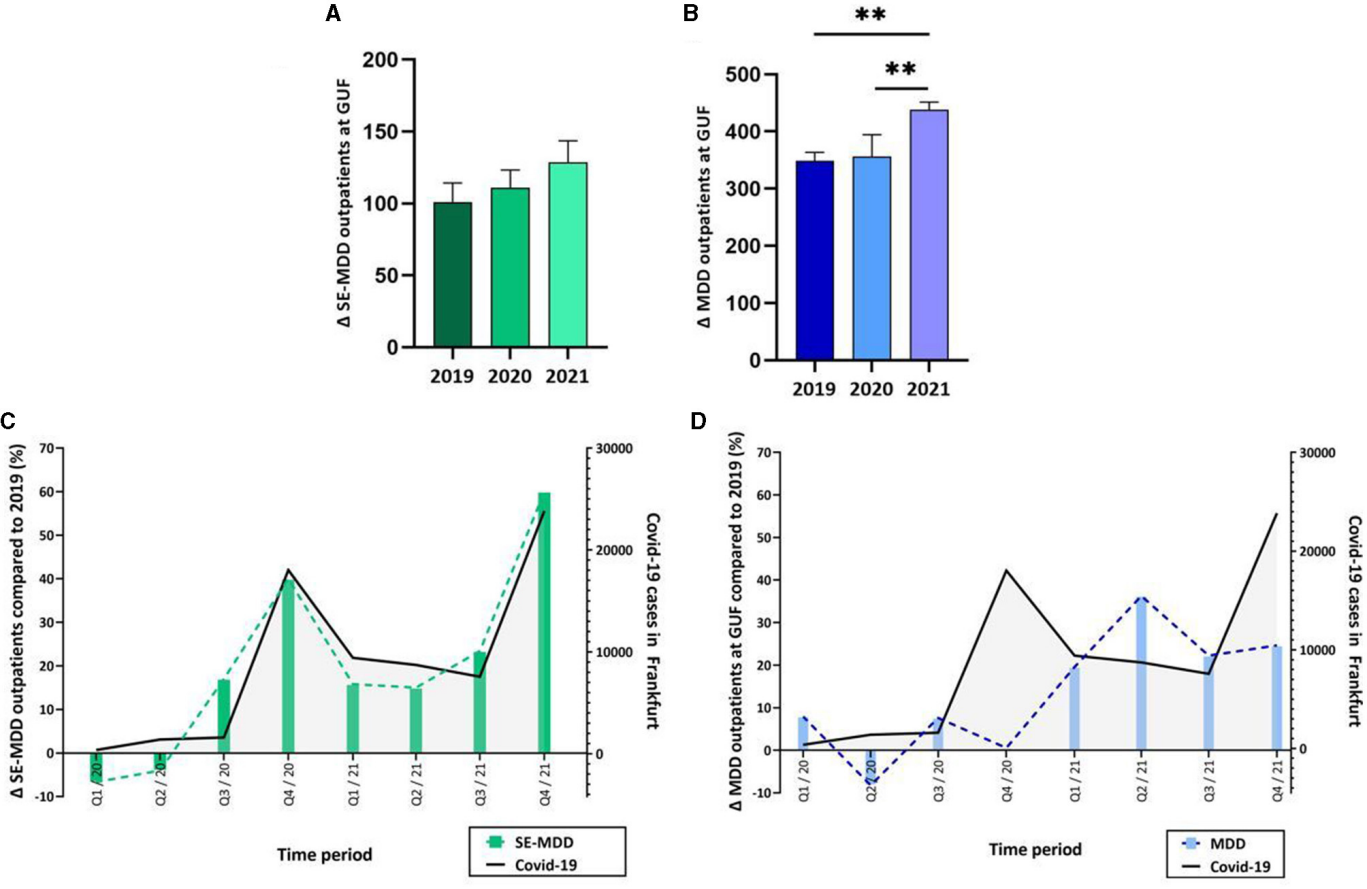
**(A)** The SE-MDD outpatient numbers at GUF did not change between the years 2019, 2020, and 2021, whereas **(B)** fewer patients with MDD were admitted to outpatient care in 2021 compared to 2020 and 2019. **(C)** More patients with SE-MDD were admitted to outpatient care at GUF beginning in the third quarter of 2020 compared to each quarter in 2019. The increase in outpatient numbers was parallel to the rise in COVID-19 rates (black line). **(D)** The percentage change of outpatients (blue) with MDD in the years 2020 and 2021 compared to 2019 showed an increase between the COVID waves. **muliplicity adj. *p* < 0.01.

We then investigated whether the decline in inpatient care was compensated by increased outpatient admissions. While the number of admissions with SE-MDD did not change between the years (Figure 4A, Table 3), we found a significant difference in outpatient numbers with MDD between 2019, 2020, and 2021 (one-way ANOVA, *F*_(2, 9)_ = 15.99, *p* = 0.0011) (Figure 4B). In particular, the *post-hoc* comparison demonstrated a significant increase between 2021 compared to 2019 (*adj. p* = 0.0017, 95% CI = −137.7,−40.26) and 2020 (multiplicity adj. *p* = 0.0030, 95% CI = −130.2,−40.26). The sex difference between male and female patients remained unchanged between the year 2019, 2020 and 2021. The quarterly Δadmission numbers in 2021 and 2020 compared to the corresponding time period in 2019 showed an increase in outpatients with SE-MDD in parallel to each COVID wave (Figure 4C). In contrast, Δadmissions with MDD declined during each wave (Figure 4D).

**Table 3:** Demographic data of outpatients at GUF in 2019, 2020, and 2021 with SE-MDD and MDD.

**Year**	**SE-MDD (*n*)**	**MDD (*n*)**	**Male (%)**	**Female (%)**
2019	404	1,396	40	60
2020	444	1,426	36	64
2021	514	1,752	36	64

## 4. Discussion

This study demonstrates a significant reduction in inpatient care for individuals with depression in Germany during the COVID-19 pandemic. Our analysis revealed a distinct decline in the number of inpatient cases during each COVID-19 wave. Consistent with these national trends, our department also observed a significant decrease in the number of MDD patients admitted to our wards. This reduction was accompanied by a shift in the ratio of planned, elective admissions to emergency admissions, and an increase in the number of outpatient admissions.

### 4.1. What caused the change in inpatient numbers during the pandemic?

During the first pandemic year of 2020, the cases of MDD increased by 53.2 million (27.6 %) (28) globally. Equally, a rise in the prevalence of depressive symptoms has been noticed in Germany (29). Despite the concerning rise in the number of patients with depression, our study revealed a notable decrease in inpatient admissions during each wave of the COVID-19 pandemic. These results build upon earlier reports of reduced psychiatric admissions, particularly for individuals with affective disorders, during the initial wave of the pandemic (22). Notably, our department observed a decrease in the number of inpatient admissions for MDD patients, while the number of admissions for individuals SE-MDD declined during each COVID wave but increased between them.

The reasons for this observation are manifold. The German government started imposing strict hygiene regulations with temporal lockdowns to prevent the spreading of the virus on the 22^nd^ of March 2020 (30). These measures did not only lead to restrictions on daily activities and personal encounters but also hampered access to healthcare facilities.

First of all, in March 2020 the German Health ministry introduced the COVID-19 Hospital Burden Reduction Act (“*COVID-19 Krankenhausentlastungsgesetz”*) (31). This act enabled compensation payments when hospitals postponed elective admissions to increase bed capacity for the care of patients with COVID-19 infections. This act was necessary to combat the pandemic but had a serious impact on the care for other serious illnesses. Psychiatric departments were obliged to keep free beds for psychiatric patients with COVID-19 infection; leading to reduced capacities for elective patients in open wards, including those suffering from depression. Second, psychiatric inpatients had to endure increased restrictions during their inpatient stay such as the ban on visits from relatives, no possibility of time outside the clinic premises, or having to wear a face mask. These measures are particularly deterrent for depressed patients, who regularly stay more than 4 weeks on the ward and already face difficulties in keeping social contact due to severe depressive symptoms. Third, the sudden onset of the pandemic led to the collapse of many supportive systems for chronically mentally ill individuals, including those with psychosis or addictive disorders. This loss of stability resulted in frequent exacerbations of symptoms, leading to an increased need for inpatient treatment (32, 33). This in turn led to reduced bed capacities for people suffering from depression. As a consequence of reduced bed capacities, patients presenting as emergency cases were more frequently admitted to closed wards. This is a familiar procedure for experienced psychiatric patients. Therefore, MDD patients might have avoided psychiatric emergency admission, stayed at home, and prolonged disease duration even more until they were forced to be admitted. In accordance, Fasshauer et al. (34) stated that the rate of involuntary patient admission increased in 2020 compared to the pre-pandemic years. The discrepancy in inpatient cases between individuals with a first depressive episode and those with recurrent depressive disorder in our department may be explained by the unfamiliarity of the former group with the admission procedure to closed wards. Additionally, younger age and lower levels of somatic comorbidity among individuals with first depressive episode compared to those with MDD may have contributed to this difference. It is possible that the fear of COVID-19 contamination was lower among SE-MDD patients than among the generally older MDD patients.

Moreover, it is not solely the government regulations that might have contributed to a worsening of care for those suffering from mental illnesses. Changes in social behavior, such as the fear to contaminate one or another when meeting friends and relatives, have potentially raised the hurdle to seeking help. Apart from the fact that reduced social contacts place a strain on mental health (35), these interactions are crucial for the detection of depressive symptoms, and the early pursuit of medical care.

### 4.2. Known sex differences in depression continue during the pandemic

It is well known, that the prevalence of depression is higher in women than men (36, 37). In line, we found higher female than male inpatient numbers. Notably, this difference remained stable after the onset of the pandemic in 2020 (28). During each wave, the decline of inpatients with both MDD and SE-MDD followed the same pattern for both sexes. Medda et al. (38) analyzed the occurrence of depressive symptoms in 1,690 adult twins from the general population in Italy and reported higher risk for depressive symptoms after the first lockdown in women. However, this sex-dependent increase was not evident in our sample of clinically diagnosed MDD and SE-MDD.

### 4.3. Partial compensation of psychiatric care by the outpatient department

Comparable to reports from other countries (39), fewer depressive outpatients were treated in our department during the initial COVID-19 wave. Nevertheless, our outpatient management markedly improved during the second wave. In 2021, we treated more patients suffering from both first and recurrent depressive episodes than in 2019. In particular, patients with recurrent depressive disorder shifted from inpatient to outpatient care, as also reported by Bollmann et al. (40) in their analysis of psychiatric patients in the German “Helios hospital network.” The advancement in technology and the growing acceptance of telemedicine by both patients and physicians (41–44) enabled consistent or even elevated outpatient capacities, compensating for reduced inpatient treatment. With growing evidence supporting the efficacy of intensive telehealth intervention (45), the COVID-19 pandemic might facilitate the use of telehealth options to improve psychiatric outpatient care.

### 4.4. Increased need and reduced treatment capacities–a vicious circle

The threat to life posed by the COVID-19-pandemic and the restrictions to daily life introduced to combat the virus have been a huge challenge for patients suffering from depression. In this context, added barriers to access psychiatric care and limited treatment capacities further deteriorated the condition. In a population-representative survey by Czaplicki et al. (46) half of the participants with depressive symptoms reported that the pandemic restrictions exacerbated their condition, with those lacking access to the psychiatric healthcare systems reporting higher symptom severity. In line, Kertzscher et al. (47) demonstrated that psychiatric outpatients with different mental illnesses who reported reduced access to medical care presented more depressive symptoms, regardless of the diagnosis.

### 4.5. The possible long-term impact of impaired access to specialized psychiatric care

Suicide represents the devastating outcome of depression. However, during the initial wave of the COVID-19 pandemic, suicide rates remained relatively stable or even decreased in 21 countries, including Germany (18). Also, Robinson et al. (48) found no change in symptom severity of preexisting psychiatric diseases in the year 2020. Nevertheless, in Italy, a country heavily affected by the pandemic, suicide attempts among psychiatric inpatients increased during 2020 pointing toward a worsening of psychiatric conditions (49). In line, Brasso et al. (50) demonstrated that MDD patients admitted to hospital after the lockdown suffered from a higher rate of suicidal ideation and more psychotic features. Furthermore, a higher antidepressant dosage and more augmentation therapy strategies were applied in those patients. These findings highlight the impact the pandemic had on depressive symptom severity (50). Also, in Japan a decline in suicide rates during the first wave was reported for children as well as for adults (51, 52), followed by a substantial increase in suicides by adults from July to October 2020 (52). This study illustrates that short-term analyses on suicide rates limited to only the first pandemic year might mask the long-term increase in suicide rates. Indeed, we suspect that in the long run, due to a decrease in inpatient numbers during every COVID-19 wave, the overall burden on patients with depression might rise eventually resulting in higher suicide rates.

### 4.6. Limitations

This study faces the following limitations: Firstly, we analyzed retrospective, highly aggregated data and had to rely on the correct use of ICD-10 codes by each hospital in Germany. Secondly, we had no information on the treatment modalities of those patients, since hospitals are not obliged to code this information for reimbursement. Third, the IneK Database does not offer data on outpatient care nor did it grant public access to data before the year 2020. We have analyzed the outpatient care in our own department. It is possible, that the outpatient care in other hospitals was differently affected.

### 4.7. Conclusion

The results of this long-term, large-scale retrospective study suggest a negative change in care for depressed patients during the pandemic. This might cause poor patient outcomes implying a major challenge for our healthcare system in the upcoming months and years. This situation calls for an improvement in mental health structures not only in Germany but worldwide, to prevent a rise in symptom burden and suicide rates, especially in light of reoccurring pandemic situations. In particular, increased funding for psychiatric hospitals, increased bed capacities and corresponding remuneration for depressive patients are needed to give financial incentives for hospitals to treat these patients as inpatients. To accomplish a long-term improvement in the outpatient sector, investments are required in the telemedical infrastructure and in improving capacities of outpatient treatment not only in hospitals but also in private practices to reduce the barrier to seeking psychiatric help. In addition, improved screening for depressive symptoms by general practitioners and adequate treatment might improve the care structure in times of highly restrained medical care. A stable and improved e-health system, stronger integration of in- and outpatient care and standardized emergency plans would provide the best way to be prepared for future pandemics and to meet the associated increased need of the psychiatric outpatient sector.

## Data availability statement

The original contributions presented in the study are included in the article, further inquiries can be directed to the corresponding author.

## Ethics statement

Ethical approval was not required for the study involving humans in accordance with the local legislation and institutional requirements. Written informed consent to participate in this study was not required from the participants or the participants' legal guardians/next of kin in accordance with the national legislation and the institutional requirements.

## Author contributions

MA: conceptualization, methodology, formal analysis, investigation, and writing-original draft. CS: methodology, validation, investigation, and writing-original draft. CU and TH: investigation. CR-L: conceptualization and writing-original draft. RG: methodology and validation. AR: conceptualization, writing-original draft, and funding acquisition. SE: conceptualization, methodology, formal analysis, investigation, writing-original draft, and project administration. All authors contributed to the article and approved the submitted version.

## References

[1] World Health Organization. Statement on the Second Meeting of the International Health Regulations (2005) Emergency Committee regarding the outbreak of novel coronavirus (2019-nCoV). (2020). Available online at: https://www.who.int/news/item/30-01-2020-statement-on-the-second-meeting-of-the-international-health-regulations-(2005)-emergency-committee-regarding-the-outbreak-of-novel-coronavirus-(2019-ncov)

[2] Böhmer MM Buchholz U Corman VM Hoch M Katz K Marosevic DV. Investigation of a COVID-19 outbreak in Germany resulting from a single travel-associated primary case: a case series. Lancet Infect Dis. (2020) 20:920–8. 10.1016/S1473-3099(20)30314-5 32422201 PMC7228725

[3] Lee J. Mental health effects of school closures during COVID-19. Lancet Child Adolesc Health. (2020) 4:421. 10.1016/S2352-4642(20)30109-7 32302537 PMC7156240

[4] Rashid S Tsao H. Effect of the COVID-19 pandemic on delayed skin cancer services. Dermatol Clin. (2021) 39:627–37. 10.1016/j.det.2021.05.015 34556252 PMC8162820

[5] Gheorghe A Maringe C Spice J Purushotham A Chalkidou K Rachet B. Economic impact of avoidable cancer deaths caused by diagnostic delay during the COVID-19 pandemic: a national population-based modelling study in England, UK. Eur J Cancer. (2021) 152:233–42. 10.1016/j.ejca.2021.04.019 34049776 PMC8530528

[6] Maringe C Spicer J Morris M Purushotham A Nolte E Sullivan R. The impact of the COVID-19 pandemic on cancer deaths due to delays in diagnosis in England, UK: a national, population-based, modelling study. Lancet Oncol. (2020) 21:1023–34. 10.1016/S1470-2045(20)30388-0 32702310 PMC7417808

[7] Kuzuu K Misawa N Ashikari K Kessoku T Kato S Hosono K. Gastrointestinal cancer stage at diagnosis before and during the COVID-19 pandemic in Japan. JAMA Netw Open. (2021) 4:e2126334. 10.1001/jamanetworkopen.2021.26334 34546368 PMC8456386

[8] Xiong J Lipsitz O Nasri F Lui LMW Gill H Phan L. Impact of COVID-19 pandemic on mental health in the general population: a systematic review. J Affect Disord. (2020) 277:55–64. 10.1016/j.jad.2020.08.001 32799105 PMC7413844

[9] Vos T Flaxman AD Naghavi M Lozano R Michaud C Ezzati M. Years lived with disability (YLDs) for 1160 sequelae of 289 diseases and injuries 1990-2010: a systematic analysis for the global burden of disease study 2010. Lancet. (2012) 380:2163–96. 10.1016/S0140-6736(12)61729-2 23245607 PMC6350784

[10] König H König HH Konnopka A. The excess costs of depression: a systematic review and meta-analysis. Epidemiol Psychiatr Sci. (2019) 29:e30. 10.1017/S2045796019000180 30947759 PMC8061284

[11] Jacobi F Höfler M Strehle J Mack S Gerschler A Scholl L. Erratum zu: psychische störungen in der allgemeinbevölkerung. studie zur gesundheit erwachsener in deutschland und ihr zusatzmodul “psychische gesundheit “(DEGS1-MH). Der Nervenarzt. (2016) 87:88–90. 10.1007/s00115-015-4458-7 26601984

[12] Lakhan R Agrawal A Sharma M. Prevalence of depression, anxiety, and stress during COVID-19 pandemic. J Neurosci Rural Pract. (2020) 11:519–25. 10.1055/s-0040-1716442 33144785 PMC7595780

[13] Salari N Hosseinian-Far A Jalali R Vaisi-Raygani A Rasoulpoor S Mohammadi M. Prevalence of stress, anxiety, depression among the general population during the COVID-19 pandemic: a systematic review and meta-analysis. Global Health. (2020) 16:57. 10.1186/s12992-020-00589-w 32631403 PMC7338126

[14] Maggi G Baldassarre I Barbaro A Cavallo ND Cropano M Nappo R. Mental health status of Italian elderly subjects during and after quarantine for the COVID-19 pandemic: a cross-sectional and longitudinal study. Psychogeriatrics. (2021) 21:540–51. 10.1111/psyg.12703 33955115 PMC8242477

[15] Jones EAK Mitra AK Bhuiyan AR. Impact of COVID-19 on mental health in adolescents: a systematic review. Int J Environ Res Public Health. (2021) 18:2470. 10.3390/ijerph18052470 33802278 PMC7967607

[16] Vindegaard N Benros ME. COVID-19 pandemic and mental health consequences: systematic review of the current evidence. Brain Behav Immun. (2020) 89:531–42. 10.1016/j.bbi.2020.05.048 32485289 PMC7260522

[17] Dubé JP Smith MM Sherry SB Hewitt PL Stewart SH. Suicide behaviors during the COVID-19 pandemic: a meta-analysis of 54 studies. Psychiatry Res. (2021) 301:113998. 10.1016/j.psychres.2021.113998 34022657 PMC9225823

[18] Pirkis J John A Shin S DelPozo-Banos M Arya V Analuisa-Aguilar P. Suicide trends in the early months of the COVID-19 pandemic: an interrupted time-series analysis of preliminary data from 21 countries. Lancet Psychiatry. (2021) 8:579–88. 10.1016/S2215-0366(21)00091-2 33862016 PMC9188435

[19] Salize HJ Rössler W Becker T. Mental health care in Germany: current state and trends. Eur Arch Psychiatry Clin Neurosci. (2007) 257:92–103. 10.1007/s00406-006-0696-9 17149540

[20] Jacobi F Becker M Bretschneider J Müllender S Thom J Hapke U. Ambulante fachärztliche versorgung psychischer Störungen. Nervenarzt. (2016) 87:1211–21. 10.1007/s00115-016-0147-4 27357454

[21] Mack S Jacobi F Gerschler A Strehle J Höfler M Busch MA. Self-reported utilization of mental health services in the adult German population–evidence for unmet needs? results of the DEGS1-mental health module (DEGS1-MH). Int J Methods Psychiatr Res. (2014) 23:289–303. 10.1002/mpr.1438 24687693 PMC6878535

[22] Fasshauer JM Bollmann A Hohenstein S Hindricks G Meier-Hellmann A Kuhlen R. Emergency hospital admissions for psychiatric disorders in a German-wide hospital network during the COVID-19 outbreak. Soc Psychiatry Psychiatr Epidemiol. (2021) 56:1469–75. 10.1007/s00127-021-02091-z 33866383 PMC8053025

[23] TeamRStudio. RStudio: Integrated Development Environment for R (2020).

[24] GraphPad Software. GraphPad Prism. 8.0.0 for Windows ed. San Diego, CA; Boston, MA.

[25] Pinheiro J Bates D DebRoy S Sarkar D Heisterkamp S Van Willigen B. Package ‘nlme'. Linear and nonlinear mixed effects models, version. (2017), p. 3.

[26] Schilling J Tolksdorf K Marquis A Faber M Pfoch T Buda S. Die verschiedenen Phasen der COVID-19-Pandemie in Deutschland: eine deskriptive analyse von januar 2020 bis februar 2021. Bundesgesundheitsblatt-Gesundheitsforschung-Gesundheitsschutz. (2021) 64:1093–106. 10.1007/s00103-021-03394-x 34374798 PMC8353925

[27] Schuppert A Polotzek K Karschau J Karagiannidis C. Effectiveness of extended shutdown measures during the 'Bundesnotbremse' introduced in the third SARS-CoV-2 wave in Germany. Infection. (2021) 49:1331–5. 10.1007/s15010-021-01713-7 34669162 PMC8526993

[28] Collaborators C-MD. Global prevalence and burden of depressive and anxiety disorders in 204 countries and territories in 2020 due to the COVID-19 pandemic. Lancet. (2021) 398:1700–12. 10.1016/S0140-6736(21)02143-7 34634250 PMC8500697

[29] Bäuerle A Teufel M Musche V Weismüller B Kohler H Hetkamp M. Increased generalized anxiety, depression and distress during the COVID-19 pandemic: a cross-sectional study in Germany. J Public Health. (2020) 42:672–8. 10.1093/pubmed/fdaa106 32657323 PMC7454766

[30] Bundesregierung. Besprechung der Bundeskanzlerin mit den Regierungschefinnen und Regierungschefs der Länder vom 22.03.2020. (2020). Available online at: https://www.bundesregierung.de/breg-de/themen/coronavirus/besprechung-der-bundeskanzlerin-mit-den-regierungschefinnen-und-regierungschefs-der-laender-vom-22-03-2020-1733248

[31] *Gesetz zum Ausgleich COVID-19 bedingter finanzieller Belastungen der Krankenhäuser und weiterer Gesundheitseinrichtungen (COVID-19-Krankenhausentlastungsgesetz)* (2020).

[32] Murthy P Narasimha VL. Effects of the COVID-19 pandemic and lockdown on alcohol use disorders and complications. Curr Opin Psychiatry. (2021) 34:376–85. 10.1097/YCO.0000000000000720 34016817 PMC8183243

[33] Vita A Barlati S. The impact of the Covid-19 pandemic on patients with schizophrenia. Eur Neuropsychopharmacol. (2022) 54:62–4. 10.1016/j.euroneuro.2021.08.003 34462180 PMC8363471

[34] Fasshauer JM Bollmann A Hohenstein S Mouratis K Hindricks G Meier-Hellmann A. Impact of COVID-19 pandemic on involuntary and urgent inpatient admissions for psychiatric disorders in a German-wide hospital network. J Psychiatr Res. (2021) 142:140–3. 10.1016/j.jpsychires.2021.07.052 34352559 PMC8417753

[35] Benke C Autenrieth LK Asselmann E Pané-Farré CA. Lockdown, quarantine measures, and social distancing: associations with depression, anxiety and distress at the beginning of the COVID-19 pandemic among adults from Germany. Psychiatry Res. (2020) 293:113462. 10.1016/j.psychres.2020.113462 32987222 PMC7500345

[36] Platt JM Bates L Jager J McLaughlin KA Keyes KM. Is the US gender gap in depression changing over time? a meta-regression. Am J Epidemiol. (2021) 190:1190–206. 10.1093/aje/kwab002 33423055 PMC8484777

[37] Jacobi F Höfler M Strehle J Mack S Gerschler A Scholl L. Psychische störungen in der allgemeinbevölkerung. Nervenarzt. (2014) 85:77–87. 10.1007/s00115-013-3961-y 24441882

[38] Medda E Toccaceli V Gigantesco A Picardi A Fagnani C Stazi MA. The COVID-19 pandemic in Italy: depressive symptoms immediately before and after the first lockdown. J Affect Disord. (2022) 298:202–8. 10.1016/j.jad.2021.10.129 34732338 PMC8557388

[39] Seo JH Kim SJ Lee M Kang JI. Impact of the COVID-19 pandemic on mental health service use among psychiatric outpatients in a tertiary hospital. J Affect Disord. (2021) 290:279–83. 10.1016/j.jad.2021.04.070 34015622 PMC9754757

[40] Bollmann A Hohenstein S Pellissier V Stengler K Reichardt P Ritz JP. Utilization of in- and outpatient hospital care in Germany during the Covid-19 pandemic insights from the German-wide helios hospital network. PLoS ONE. (2021) 16:e0249251. 10.1371/journal.pone.0249251 33765096 PMC7993839

[41] Cosić K Popović S Šarlija M KesedŽić I. Impact of human disasters and COVID-19 pandemic on mental health: potential of digital psychiatry. Psychiatr Danub. (2020) 32:25–31. 10.24869/psyd.2020.25 32303026

[42] Chen JA Chung WJ Young SK Tuttle MC Collins MB Darghouth SL. COVID-19 and telepsychiatry: early outpatient experiences and implications for the future. Gen Hosp Psychiatry. (2020) 66:89–95. 10.1016/j.genhosppsych.2020.07.002 32750604 PMC7347331

[43] Di Carlo F Sociali A Picutti E Pettorruso M Vellante F Verrastro V. Telepsychiatry and other cutting-edge technologies in COVID-19 pandemic: Bridging the distance in mental health assistance. Int J Clin Pract. (2021) 75. 10.1111/ijcp.13716 32946641 PMC7536971

[44] O'Brien M McNicholas F. The use of telepsychiatry during COVID-19 and beyond. Ir J Psychol Med. (2020) 37:250–5. 10.1017/ipm.2020.54 32434596 PMC7411439

[45] Levinson CA Spoor SP Keshishian AC Pruitt A. Pilot outcomes from a multidisciplinary telehealth versus in-person intensive outpatient program for eating disorders during versus before the Covid-19 pandemic. Int J Eat Disord. (2021) 54:1672–9. 10.1002/eat.23579 34245028

[46] Czaplicki A Reich H Hegerl U. Lockdown measures against the spread of the COVID-19 pandemic: negative effects for people living with depression. Front Psychol. (2022) 13:789173. 10.3389/fpsyg.2022.789173 35185723 PMC8854217

[47] Kertzscher L Kohls E Baldofski S Moeller R Schomerus G Rummel-Kluge C. Managing the COVID-19 pandemic in people with mental disorders: an exploratory telephone interview study in a psychiatric outpatient department. Compr Psychiatry. (2022) 116:152313. 10.1016/j.comppsych.2022.152313 35429763 PMC8993418

[48] Robinson E Sutin AR Daly M Jones A. A systematic review and meta-analysis of longitudinal cohort studies comparing mental health before versus during the COVID-19 pandemic in 2020. J Affect Disord. (2022) 296:567–76. 10.1016/j.jad.2021.09.098 34600966 PMC8578001

[49] Berardelli I Sarubbi S Rogante E Cifrodelli M Erbuto D Innamorati M. The impact of the COVID-19 pandemic on suicide ideation and suicide attempts in a sample of psychiatric inpatients. Psychiatry Res. (2021) 303:114072. 10.1016/j.psychres.2021.114072 34256349 PMC8543059

[50] Brasso C Cisotto M Del Favero E Giordano B Villari V Rocca P. Impact of COVID-19 pandemic on major depressive disorder in acute psychiatric inpatients. Front Psychol. (2023) 14:1181832. 10.3389/fpsyg.2023.1181832 37303894 PMC10249995

[51] Isumi A Doi S Yamaoka Y Takahashi K Fujiwara T. Do suicide rates in children and adolescents change during school closure in Japan? the acute effect of the first wave of COVID-19 pandemic on child and adolescent mental health. Child Abuse Negl. (2020) 110:104680. 10.1016/j.chiabu.2020.104680 32847679 PMC7443207

[52] Tanaka T Okamoto S. Increase in suicide following an initial decline during the COVID-19 pandemic in Japan. Nat Hum Behav. (2021) 5:229–38. 10.1038/s41562-020-01042-z 33452498

